# A survey on radiation protection awareness at various hospitals in Karachi, Pakistan

**DOI:** 10.1016/j.heliyon.2022.e11236

**Published:** 2022-10-27

**Authors:** Mishkat Ali Jafri, Salman Farrukh, Roohi Zafar, Nasir Ilyas

**Affiliations:** a Institute of Business Administration, Karachi, Pakistan; b Atomic Energy Medical Centre, Jinnah Postgraduate Medical Centre, Karachi, Pakistan; c NED University of Engineering and Technology, Karachi, Pakistan; d University of Karachi, Karachi, Pakistan

**Keywords:** Radiation protection, Karachi, Knowledge level, Medical radiological practitioners, Medical physicists, Medical radiation technologists

## Abstract

**Purpose:**

To assess the awareness level of radiation protection among the radiation workers (i.e. medical radiation technologists, medical physicists, and medical radiological professionals) at the selected radiology, nuclear medicine, and radiotherapy facilities in Karachi, Pakistan.

**Material and methods:**

This survey was carried out in Karachi which has the largest number of hospitals, including radiology, nuclear medicine, and radiotherapy facilities in all Pakistan. In this double-blind survey, a questionnaire was designed and distributed to one hundred and sixty five (165) medical radiation workers at their respective workplaces. These radiation workers included the medical radiation technologists, medical physicists, and medical radiological practitioners. These radiation workers had varying experiences, training records, education, and fields of specialization. Out of these total 165 respondents, 84 belonged to the radiology facilities, 20 to nuclear medicine facilities, and 61 to radiotherapy facilities. The educational level was classified as less than 16 years, and equal to or greater than 16 years. Similarly, the training was classified as “attended” or “never attended” and the experience as less than five years, between 5 and 10 years, and greater than ten years. The data was processed through SPSS (v.20) against a significance level (P ≤ 0.05).

**Results:**

The statistical analysis of the survey indicates that the radiation workers in radiology, nuclear medicine, and radiotherapy facilities in Karachi have limited awareness of radiation protection issues. The overall outcome of the survey also concluded that the awareness regarding radiation effects, radiation warning signs, and annual dose limit is optimum. However, the response to the questions related to patient protection remained unsatisfactory.

**Conclusion:**

This study showed that most of the radiation workers who participated in the survey lacked appropriate awareness of radiation protection measures. The radiation workers did show a better understanding of basic radiation protection parameters, such as the annual dose limit and radiation effects. However, the radiation workers needed an overall improvement in radiation protection awareness, particularly, related to patient protection. This awareness and knowledge should be improved through systematic and periodic trainings.

## 1 Introduction

Applications of the ionizing radiation (hereinafter called radiation) have increased in many fields of life. Medicine is one of the leading examples where radiations are used to diagnose and treat patients. Among all the applications of radiation, medical applications (i.e. radiology, nuclear medicine, and radiotherapy) are a source of a low to high level of radiation received by the humans. Therefore, radiation protection measures must be taken for the patients, workers, and public because the radiation doses can vary from low to very high in radiation medicine.

Considering the well-known radiation effects on human body, the International Atomic Energy Agency (IAEA) has issued “Radiation Protection and Safety of Radiation Sources: International Basic Safety Standards” (GSR Part 3) which addresses, among other items of radiation safety and protection, the requirements of training on radiation protection for all types radiation workers [1].

The training and re-training on radiation protection topics is a tool and mean to protect themselves (i.e. radiation workers), co-workers, the public, and patients from the harmful effects of radiation. After the issuance of the IAEA GSR Part 3 (which is a requirement level publication), the IAEA issued a Safety Guide “Radiation Protection and Safety in Medical Uses of Ionizing Radiation (SSG-46)”, which was a specific guidance publication addressing the practical implementation of the safety standards and parameters within a medical radiation facility and practice [2]. This guide (SSG-46) strongly recommends the need of training for various categories of radiation workers like the medical radiological professionals, medical physicists, medical radiation technologists and radiation protection officers. The need of trainings on radiation safety topics have also been explained and addressed in several other IAEA publications [3].

The IAEA member states widely use these IAEA publications according to their own situation. In case of Pakistan, the IAEA safety standards, regulations and the regulatory guides are applicable and these publications address the need and details of training on radiation safety [3]. Therefore, having appropriate training on radiation safety is not only beneficial for working conditions but it is also a regulatory requirement in Pakistan.

Considering the importance of radiation safety trainings and knowledge level, several researchers have conducted surveys at medical facilities to measure the knowledge of radiation workers regarding radiation safety aspects. A study was carried out in Turkey among one hundred and one (101) healthcare personnel, and the study concluded that the radiation workers were not adequately aware of the radiation safety requirements [4]. Another study in Malaysia assessed that the radiation safety awareness among the nuclear medicine nursing staff was at a moderate level [5]. The authors of the study recommended that a national study should be conducted to assess and to increase knowledge and awareness among all nuclear medicine nurses in Malaysia. A relatively large study of seven hundred and eighty (780) radiation workers in Italy showed a crucial need of awareness of radiation safety among radiation workers [6]. Another survey which was conducted in Italy to assess the dose levels in radiology facilities concluded that the radiology physicians (including the residents), radiography students, and medical students have minimal awareness about radiation protection. They also identified a knowledge gap concerning actual radiation doses of daily radiological examinations [7]. The study recommended that undergraduate and postgraduate teaching and training should be effectively designed and implemented at the facilities. Another study, to assess the knowledge of radiation dose and risk incurred in common radiology examinations among radiology residents, fellows, staff radiologists and technologists in Ottawa, Canada, showed the variable level of knowledge about radiation dose and risk among the ninety two (92) participants and concluded that overall situation was not satisfactory [8]. In a study conducted at Hamadan city, Iran, the response of seventy one (71) participants showed that overall condition of radiation protection awareness was good but there was still a need of improvement through further trainings [9]. In a study conducted at Taif city, Saudi Arabia for seventy five (75) radiographers, the awareness level was found to be satisfactory and the radiographers were found following appropriate safety standards during their practices [10]. Another study was conducted among eighty two (82) participants in Norway to explore if the perception of radiologists and radiographers on referral practice differs from that of referring clinicians, and to see if knowledge of radiation issues and referral guidelines differ between these groups. The study concluded that all groups have a potential to improve their radiation protection knowledge [11]. In a study, the authors evaluated the level of knowledge and awareness among one hundred and twenty (120) radiology personnel working in seven public hospitals in Johor, Malaysia, concerning Computed Tomography (CT) technology and radiation doses based on a set of questionnaires. The study concluded that there was a considerable variation among the answers of the participants and they were not well versed with CT optimization techniques [12]. A cross-sectional study was conducted among the one hundred and ninety seven (197) radiographers in various hospitals at the UAE in 2017. The study concluded that the practices of radiographers were unsatisfactory about radiation safety standards [13]. One study of two hundred and fourteen (214) participants at Bangkok, Thailand revealed that there was a reasonable level of awareness but a relative lack of knowledge about radiation hazards and protection among anesthesia personnel and surgical subspecialists [14]. In a study, which took place in Cyprus for the radiographers, it was concluded that the radiation safety awareness was at good level but further improvement in understanding the dose limits was needed [15]. One study in Turkey in which ninety two (92) participants took part concluded that their level of knowledge about ionizing radiation and doses in radiological examinations was very weak [16].

Several such studies have been conducted in different cities of Pakistan as well. In order to determine the radiation safety awareness level of medical radiation technologists in Peshawar, a survey was conducted among forty one (41) medical radiation technologists. In the findings of this study, the researchers identified a solid need to improve radiation safety trainings and awareness [17]. Another study observed the radiation protection practices of the twenty nine (29) radiation workers in radiology in Muzaffarabad city. This study found that there was substantial need to improve radiation safety awareness among the users of radiation sources [18]. A separate study, conducted in a public sector hospital in the province of Khyber Pakhtoonkhwa also agreed with the statement [19].

In contrast to these studies, one study found that radiation safety awareness was at the optimum level, however, the radiation safety practices and gadgets were inappropriate due to lack of financial resources [20]. Several other studies show that there have been many training courses and seminars, which would undoubtedly improve radiation safety awareness gradually with time among the radiation workers [21, 22].

It can be summarized that radiation safety training and knowledge are directly linked with the IAEA’s safety standards and the Pakistan Nuclear Regulatory Authority (PNRA) regulation. In particular, the PNRA Regulations on Radiation Protection (PAK/904), based on IAEA Safety Standards GSR Part 3, has made firm recommendations on the trainings and retraining of radiation workers. A vast majority of surveys show that the awareness of radiation safety is a global challenge and the local conditions of Pakistan are no exception. Therefore, radiation safety trainings and awareness need to be improved to minimize the chances of harmful effects caused by the radiation exposure.

The current study has been designed to analyze the awareness of radiation safety at the medical facilities where radiations are used i.e. radiology, nuclear medicine, and radiotherapy. The study was conducted at over fifty medical facilities in Karachi. In contrast with other surveys, this study incorporates different fields of radiation medicine i.e. radiology, nuclear medicine, and radiotherapy that are in accordance with the IAEA safety standard and the national regulations. This study also incorporates the impact of education level and any level of training on radiation safety among various radiation workers.

## 2 Materials and methods

The best way to assess the awareness level of any area of interest is to perform surveys. We conducted a double-blind survey with particular and standard questions to conclude our results. In this regard, a questionnaire was designed and distributed among one hundred and sixty five radiation workers with varying experiences, training records, education, and fields of specialization.

The data was obtained from the survey of medical radiation technologists, medical physicists and medical radiological professionals at different hospitals in Karachi from Jan 1st to Dec 31st, 2020. This data was collected from over fifty small and large hospitals having either radiology, nuclear medicine, or radiotherapy facilities. Before this survey, the participants were informed that the data would be stored in a database and used for research purposes only. Participation in the survey was voluntary and completely anonymous. A total of one hundred and sixty five (165) workers joined this survey that is classified as: radiology = 84 (50.9%), nuclear medicine = 20 (12.1%), and radiotherapy = 61 (37.0%).

An attempt was also made to increase the difficulty of questions as the questionnaire progressed gradually. Therefore, the initial questions were about the fundamental concepts and the last questions were about the regulations on radiation protection. The survey questionnaire was divided into following two parts (see Table 1).

Section 1: A consent included the demographic information of the participants (experience, qualification, training, and specialization).

Section 2: Another consent included the knowledge about radiation effect, shielding material, exposure effect, annual dose limit, radiation limit in the controlled area, warning sign, medical exposure, and patient protection.

All questions in section 2 were formulated as the multiple-choice questions with 2–5 appropriate options. The un-attempted answers were also counted as the wrong one.

For Statistical analysis, the F statistics ANOVA test was performed by using software with a significance level (P ≤ 0.05) (SPSS version 20.0, www-01.ibm.com/software/analytics/SPSS).

**Table 1:** The questionnaire.

1. Field of specialization: a. Radiology. b. Nuclear Medicine. c. Radiotherapy	2. Education: a. Less than 16 years. b. Equal or more than 16 years.
3. Have you attended any course/workshop on radiation protection? a. Yes b. No	4. Work experience: a. Less than five years a. 5–10 years. b. Greater than ten years.
5. Radiation produces harmful effects on the body: a. Yes b. No c. Do not know	6. Which of the following is the best shielding material for gamma/x-rays? a. Aluminum. b. Lead. c. Concrete. d. Don’t know
7. Low-level exposure produces the following effects in the body: a. Stochastic effect. b. Deterministic effect. c. Both. d. Don’t know	8. The annual dose limit for a radiation worker is: a. 1 mSv. b. 6 mSv. c. 20 mSv. d. Don’t know.
9. The limit of radiation in the controlled area is: 1 mSv. 6 mSv. 20 mSv. Don’t know.	10. Which of the following is a radiation sign? a. b. c. d.
11. Radiation exposure to which of the following can be classified as “medical exposure”? a. Exposure to patients. b. Exposure to comforter/Carer. c. Exposure in biomedical research. d. All of the above. e. Don’t know.	12. The prime responsibility of protection of patients lies with: a. Physician. b. Radiation protection officer. c. Technologist. d. All of the above. e. Don’t know.

## 3 Results and discussion

A total of 165 radiation workers responded to the issued questionnaires and the outcome of the survey was statistically categorized and presented in Tables 2, 3, 4, and 5. The responses to the questionnaire were distributed showing the mean score, standard deviations (S.D), and p-value according to different categories. Table 2 gives the statistics among the specialization fields (i.e. radiology, nuclear medicine, and radiotherapy). Table 3 gives statistics among educational levels (in terms of years). Table 4 gives statistics among the trained and partially trained workers, and Table 5 gives the statistics among experience levels (in terms of years: less than five years, between 5 and 10 years, and greater than ten years).

**Table 2:** Responses according to field of specialization.

Specialization	Radiology (N = 84)		Nuclear Medicine (N = 20)		Radiotherapy (N = 61)		P Value
Responses	Mean	S.D	Mean	S.D	Mean	S.D	
**Radiation effect**	0.964	0.186	0.95	0.223	0.951	0.218	0.911
**Shielding material**	0.892	0.311	1.000	0.000	0.967	0.179	0.093
**Low level Exposure effect**	0.25	0.435	0.55	0.510	0.737	0.443	0.000∗∗
**Annual dose limit**	0.892	0.311	0.850	0.366	0.902	0.300	0.813
**Controlled area radiation limit**	0.512	0.503	0.1	0.307	0.049	0.218	0.000∗∗
**Warning sign**	0.905	0.295	0.85	0.366	0.967	0.179	0.177
**Medical exposure**	0.440	0.499	0.1	0.307	0.180	0.387	0.000∗∗
**Responsibility of patient protection**	0.167	0.179	0.05	0.224	0.328	0.179	0.23

S. D = Standard deviation, ∗ = Significant, ∗∗ = Highly significant.

**Table 3:** Responses according to education level.

Education	Less than 16 Years (N = 67)		Equal and greater than 16 Years (N = 98)<>		P-Value
Responses	Mean	S. D	Mean	S. D	
**Radiation effect**	0.955	0.208	0.959	0.198	0.902
**Shielding material**	0.881	0.326	0.969	0.173	0.025∗
**Low-level Exposure effect**	0.403	0.494	0.510	0.502	0.177
**Annual dose limit**	0.881	0.326	0.898	0.304	0.727
**Controlled area radiation limit**	0.254	0438	0.316	0.467	0.388
**Warning sign**	0.895	0.308	0.938	0.241	0.314
**Medical exposure**	0.268	0.446	0.326	0.471	0.430
**Responsibility of patient protection**	0.104	0.308	0.102	0.304	0.960

**Table 4:** Responses on professional training.

Specialized Training	Yes (N = 108)		No (N = 57)		P-Value
Responses	Mean	S. D	Mean	S. D	
**Radiation effect**	0.963	0.189	0.946	0.225	0639
**Shielding material**	0.935	0.247	0.929	0.257	0896
**Low-Level Exposure effect**	0.518	0.502	0.368	0.486	0.067
**Annual dose limit**	0.953	0.211	0.772	0.423	0.000∗∗
**Controlled area radiation limit**	0.277	0.449	0.316	0.469	0.612
**Warning sign**	0.953	0.211	0.859	0.350	0.033∗
**Medical exposure**	0.277	0.449	0.351	0.481	0.334
**Responsibility of patient protection**	0.071	0.263	0.158	0.368	0.093

**Table 5:** Responses on working experience.

Experience	Less than 5 Years (N = 50)		Between 5 and 10 years (N = 48)		Greater than 10 years (N = 67)		P-Value
Responses	Mean	S. D	Mean	S. D	Mean	S. D	
**Radiation effect**	0.960	0.198	1.00	0.00	0.925	0.265	0.148
**Shielding material**	0.960	0.198	0.917	0.279	0.933	0.250	0.657
**Low level Exposure effect**	0.42	0.498	0.521	0.504	0.467	0.500	0.609
**Annual dose limit**	0.88	0.328	0.916	0.279	0.891	0.313	0.797
**Controlled area radiation limit**	0.300	0.462	0.292	0.459	0.291	0.455	0.982
**Warning sign**	0.900	0.303	0.917	0.279	0.921	0.270	0.723
**Medical exposure**	0.320	0.471	0.312	0.468	0.303	0.461	0.903
**Responsibility of patient protection**	0.020	0.141	0.083	0.279	0.103	0.305	0.017∗

A significant finding of the responses shows that the participants have better knowledge about the technical questions than the regulatory ones. More than 90% of the respondents answered the first question in almost all cases correctly. However, the rate dropped below 20% in most cases when they were asked about regulatory questions (like the prime responsibility of patient protection). Two exceptions, however, can be observed in the case of annual dose limits and radiation warning signs (probably due to the easy and straightforward nature of these questions).

A discussion on the responses and results is given below and an attempt has been made to explain any crucial findings in mean value and the P-value of the responses in the light of experience feedback and other published literature.

Table 2 presents the responses of the workers according to their field of specialization. This field of specialization has been made in accordance with the IAEA safety standards [1]. Our data shows that in five out of eight questions, the results were not statistically significant among all types of specializations. The response to three questions: (i) low-level radiation effects (P = 0.00), (ii) dose limits in a controlled area (P = 0.00), and (iii) definition of medical exposure (P = 0.00) were found to be statistically significant. The radiotherapy workers responded better in the case of low-level radiation effects (73.7% correct) followed by the nuclear medicine (55.0% correct) and radiology (25% correct). Considering this question, an easy and straightforward one, the inadequate response of workers shows poor training or awareness of radiation protection, particularly in radiology. In contrast, radiology workers responded better to the questions related to the dose limit at the controlled area (51.2% correct) and the definition of medical exposure (44.0% correct).

As far as the mean values of the correct answers are concerned, a clear trend can be observed, which shows that the difficulty of questions remained uniform for all three categories. The radiotherapy workers provided the least correct response for dose limits at the controlled area (4.9% correct), followed by the nuclear medicine workers about the prime responsibility of patient protection (5% correct), the definition of medical exposure (10% correct), and the dose limit of controlled area (10% correct). The radiology workers provided the lowest response for the prime responsibility of patient protection (16.7% correct). As far as the highest score is concerned, the radiology workers scored the highest for radiation effects (96.4% correct), the nuclear medicine workers for the shielding material (100% correct), and the radiotherapy workers for the shielding material and the warning signs (both 96.7% correct).

Table 3 represents the impact of education on the awareness level of radiation workers. As a general perception, better result was expected from the more educated people regarding the number of academic years.

In Pakistan, an underlying understanding is that medical physicists and radiological medical practitioners possess at least graduate-level education, whereas medical radiation technologists generally possess undergraduate education. This attribute is also mentioned in the PNRA Regulations on Radiation Protection (PAK/904). Therefore, education of fewer than 16 years mostly means a medical radiation technologist, whereas education of 16 or more years means a medical physicist or radiological medical practitioner.

Regarding the impact of education on the responses, the only statistically significant response was about the shielding material (P = 0.025). Other considerable P-values were observed for the effect of low-level radiation (P = 0.177), the radiation warning sign (P = 0.314), and the dose limit in the controlled area (P = 0.388).

Regarding the mean values of the responses, the percentage of correct questions generally drops from high to low as the questionnaire progresses. The undergraduate score is the highest (95.5% correct) for the radiation effects and lowest (10.4% correct) for the prime responsibility of patient protection. In comparison, the graduates' score is the highest (96.9% correct) for he shielding material and lowest (10.2% correct) for the prime responsibility of patient protection.

In almost all cases, workers with better education scored higher than those with less education (except for the prime responsibility of patient protection, in which the scores for undergraduates and graduates are 10.4% and 10.2%, respectively).

Table 4 represents the impact of training on the awareness level of radiation workers. It is anticipated that radiation workers who have received trainings on radiation protection topics should have better knowledge than those who have never attended such trainings.

The statistically significant outcome was observed for the two cases i.e. the annual dose limits (P = 0.00) and the radiation warning sign (P = 0.033). Other considerable P values are for the effects of low-level radiation (P = 0.067) and the prime responsibility of patient protection (P = 0.093).

The correct answer’s mean value follows a similar general trend: i.e. from high to low as the questionnaire proceeds. The workers who have attended training on radiation protection have scored the highest for radiation effects (96.3% correct) and the lowest for the prime responsibility of patient protection (7.1% correct). In this case, the person who has never attended a training course scored highest for radiation effects (94.6% correct) and lowest for the prime responsibility of patient protection (15.8% correct).

It is also noted that the workers who have received training on radiation protection provided better responses of five out of eight questions. Whereas the workers with no training scored better for the dose limit of controlled area (31.6% correct), the definition of the medical exposure (35.1% correct), and the prime responsibility of patient protection (15.8% correct).

Table 5 shows the impact of the experience of the workers on radiation protection awareness. The experience has been classified as less than 5 years, between 5 and 10 years, and greater than 10 years. It is anticipated that workers with higher experience should better understand radiation protection.

The outcome was statistically significant in the prime responsibility of patient protection (P = 0.017). Another significant P-value for radiation effects (P = 0.148). The rest of the data and responses show that there has been no significant impact of the experience on the awareness of radiation protection.

The highest mean value of the low experience of workers is for the radiation effects and the shielding material (96.0% correct). At the same time, the lowest score was for the prime responsibility of patient protection (2.0% correct). In the case of 5–10-year experience, the workers scored highest for the radiation effects (100% correct) and lowest for the prime responsibility of patient protection (8.3% correct). In the case of higher experience (i.e. greater than 10 years), the highest score is for the shielding material (93.3% correct) and the lowest for the prime responsibility of patient protection (10.3%).

It is interesting to note that except for two out of eight questions (i.e. the warning sign and the prime responsibility of patient protection), the workers with higher experience (i.e. greater than 10 years) scored lower than those with less experience. This is probably because workers become complacent over time and do not remain eager to update or maintain the desired level of knowledge in radiation protection.

The results obtained in this section are not satisfactory and similar to the national and international published data, as discussed in the introduction section [4, 5, 6, 7, 8, 9, 10, 11]. This is an exciting finding, which leads us to set some potential work in the future. For example, there might be a need to survey at the management level to assess their concerns about radiation protection.

## 4 Conclusion

Our findings indicate that most of the radiation workers in medical settings, regardless of their field of expertise, experience, training, and educational level, lack a good understanding of radiation protection awareness. In particular, the radiation workers show poor understanding about the questions related to medical exposures (i.e. the definition of medical exposure and the prime responsibility of patient protection). However, the workers better understood some easy and straightforward questions like the radiation effects, annual dose limits, and warning signs. A significant observation of the responses is that the participants have better knowledge about technical questions than that of regulatory-related ones. The lowest score among all cases was with mean = 0.020 when the question on the prime responsibility of patient protection was asked from the workers having experience of less than 5 years. Whereas, the question about the shielding material was responded correctly by all participants in the case of nuclear medicine. In addition to this, all the participants, having experience between 5 and 10 years, gave correct answer for the question about the effects of radiation. This situation demands corrective actions and tailored training programs at multiple levels (i.e. managerial and workers level) to enhance the awareness of radiation protection at a mass level.

## Declarations

### Author contribution statement

Mishkat Ali Jafri; Salman Farrukh: Conceived and designed the experiments; Performed the experiments; Analyzed and interpreted the data; Contributed reagents, materials, analysis tools or data; Wrote the paper.

Nasir Ilyas: Conceived and designed the experiments; Performed the experiments; Analyzed and interpreted the data; Contributed reagents, materials, analysis tools or data.

### Funding statement

This research did not receive any specific grant from funding agencies in the public, commercial, or not-for-profit sectors.

### Data availability statement

Data included in article/supp. material/referenced in article.

### Declaration of interest’s statement

The authors declare no conflict of interest.

### Additional information

No additional information is available for this paper.
